# Revealing good deeds: disclosure of social responsibility in competitive markets

**DOI:** 10.1007/s10683-022-09752-z

**Published:** 2022-03-23

**Authors:** Sören Harrs, Bettina Rockenbach, Lukas M. Wenner

**Affiliations:** Department of Economics, University of Cologne, Albertus Magnus Platz, 50923 Cologne, Germany

**Keywords:** Social responsibility, Market experiments, Disclosure, Consumer behavior, C92, D90, D62, M14

## Abstract

We experimentally study competitive markets with socially responsible production. Our main focus is on the producers’ decision whether or not to reveal the degree of social responsibility of their product. Compared to two benchmark cases where either full transparency is enforced or no disclosure is possible, we show that voluntary and costless disclosure comes close to the full transparency benchmark. However, when the informational content of disclosure is imperfect, social responsibility in the market is significantly lower than under full transparency. Our results highlight an important role for transparent and standardized information about social externalities.

**Supplementary Information:**

The online version contains supplementary material available at 10.1007/s10683-022-09752-z.

## Introduction

The social responsibility (SR) of products and their production processes is a central topic of many public controversies. Prime examples are the accusation of deficiencies in working conditions (e.g., in garment factories), damaging environmental effects (e.g., in CO$_2$ emissions), or a lack of sustainability in production (e.g., in using non-recyclable plastics). While there seems to be an increasing awareness of consumers about the wider social impact of the purchase of many products, suggestive evidence points at overall levels of SR in markets being fairly low.^1^

The existing literature, however, shows that it is not that consumers do not care at all about SR, but instead exhibit a significant increase in their willingness-to-pay for these types of products.^2^ Then how does this square with the idea that in many industries SR products often only have very small market shares? If consumers care about the social responsibility of the purchased products, this should be a relevant dimension for firms to compete in, and firms offering SR products should have a competitive advantage. This argument, however, relies on the assumption that consumers can assess the social impact of a product. Yet, typically SR is not a directly visible characteristic of the product, but one that needs to be actively revealed by the seller, like, for example, the working conditions in the production process. Although in some domains revelation of the SR in production through producer declarations seems an appropriate solution (e.g., declaration of a piece of furniture to be free of tropical timber), in many cases incentives for providing non-truthful specifications are strong and hardly verifiable (e.g., due to complex opaque production chains). In such cases, labels certifying SR in production may be a powerful solution. The multiplicity of labels has tremendously increased in recent years. Some labels address single issues, like the energy efficiency class of an electric appliance, while others cover multiple facets. For example, fair trade as well as animal welfare are two dimensions of social responsibility where a large number of different labels for seemingly similar issues exist.^3^ Arguably, if the externalities of products are measured in various different ways by considering various different facets, this considerably lowers the informational content of those labels for consumers.

In this paper, we experimentally study the emergence of SR in competitive markets where SR is not a directly visible characteristic of the product, but information about the SR of the product can be voluntary provided by its seller. In the experiment, our proxy for SR is a monetary transfer to the charity *unicef*. Each seller chooses the price at which he offers the product, and an amount (also referred to as “donation” below) to be transferred to *unicef* in case he sells the product to a consumer. Donations do not influence the consumer’s monetary benefit from the product, but the vast majority of participants in our experiment consider *unicef* a charity worth supporting. Higher donations incur higher monetary costs for the seller.^4^

To get a nuanced picture we study various scenarios in our experimental treatments. Our benchmark cases are that customers are either completely ignorant (No Info) or fully informed (Full Info) about the donation associated with the product (i.e. the SR of the product). The degree of information (either none or full) is externally enforced and neither producers nor customers can change this. The Full Info case mirrors a situation in which, for example, regulation requires an unambiguous declaration of the SR characteristic of the product, e.g. the specification of the energy efficiency class of a light bulb or a fridge.

Three further Choice treatments mimic situations in which producers can choose to reveal the SR information of their product to customers (e.g., by certification through a label), but revelation is potentially imperfect. The Choice treatments vary the degree of the imperfection of this revelation. In treatment Choice-100 customers unambiguously see the information whenever a producer decides to reveal the SR information. This resembles the case of a (standardized) label and customers that are aware of what this label certifies (e.g., declaration of the absence of tropical timber). In treatments Choice-85 and Choice-60 the producer also decides whether or not to reveal the SR information, but - in case of a revelation decision - both the revelation decision and the revealed SR information may not reach the consumer (in Choice-85 there is a 85 percent chance that the revelation decision and SR information reaches consumers and there is a 60 percent chance in Choice-60). These two treatments are designed to map situations in which the producer faces an uncertainty whether his SR information reaches the consumers. Reasons for this uncertainty may be that consumers do not get the informational content of the provided information, e.g., due to a lack of standardization or limited attention of consumers. Hence, in treatments Choice-85 and Choice-60, a consumer facing non-disclosed information does not know whether the producer chose not to disclose or whether he disclosed, but the information was not transmitted. This means that while strategically sophisticated buyers should interpret undisclosed donations in Choice-100 as zero donations, this is no longer the case in Choice-85 and Choice-60. The unreliability of disclosure provides strategic incentives to producers to offer products with a lower level of social responsibility compared to Choice-100 where buyers can rely on non-disclosure being fully reliable.

In a nutshell, our results show that disclosure of SR information has large and positive effects compared to a regime where producers are unable to disclose the social responsibility of their products to consumers (No Info). We find that when voluntary disclosure provides unambiguous information for customers (Choice-100), the level of social responsibility comes close to the outcome under full and externally enforced disclosure (Full Info). While this is, in general, positive news when trying to establish more socially responsible markets, our results for the cases where disclosure is imperfect show the limitations of such an approach. When producers cannot be sure that the provided SR information has full informational content for consumers (Choice-85 and Choice-60), SR in production is significantly lower compared to Full Info. Yet, since our data does not provide an unambiguous statistical difference between the three choice treatments, the sensitivity of social responsibility to changes in the disclosure probability cannot be precisely determined. Furthermore, we show that producers benefit from more opaque information environments via higher profits. They produce products at lower cost, while selling them with a higher mark-up, taking advantage of the reduced competition in the market.

Our results have important implications for the understanding of markets with a social externality. In particular, they can provide guidance for policy makers and regulators interested in increasing socially responsible production or products. This is true in particular for environments where there is scope for “demand-driven” social responsibility, i.e., markets where consumers care about the social impact of the product they purchase and have enough influence to affect sellers’ production choices. Then, competitive markets can deliver high levels of social responsibility, but only when transparency and clarity of the latter is enforced. Since such strict policies may be impractical to implement, we show that voluntary disclosure, which requires little regulatory intervention, seems to be a viable alternative to also achieve high levels of SR. However, our results also indicate that this is not true if producers can disclose the social responsibility of their products only in a way that is unreliable. This provides a rationale for coherent labels with standardized criteria. In many relevant industries, however, this is not the case, as the examples provided above show. Our results are consistent with markets for such products displaying low production standards for a majority of the products sold. They also provide a clear reason why neither forced disclosure nor more informative and standardized labels are in the interest of the producers: Producers benefit from the reduced transparency through higher profits, and thus have strong incentives to actively oppose such labels.

## Related literature

Our paper adds to an increasing body of literature in economics which studies the determinants of moral behavior, see e.g., Irlenbusch and Villeval (2015) for an overview. More specifically, we focus on the emergence of social responsibility in markets. To our knowledge, there is no paper within the experimental literature which explicitly analyzes, as our paper does, the firms’ decision whether or not to reveal the level of social responsibility of the offered product.

Bartling et al. (2015) study a laboratory market where products with and without an externality, represented by the earnings of a third party, can be traded. They find that behavior in those markets reflects a concern for social responsibility, demonstrated through market shares for the socially responsible product of up to 50%. Using the same basic paradigm, Bartling et al. (2019) show that these results do not depend on whether social harm is concentrated on a single subject or diffused among many, while Bartling et al. (2020) show that an exogenous increase in consumer incomes increases social responsibility in the market. Using, like us, a charity rather than another lab participant representing the externality, Rode et al. (2008) also provide evidence that consumers are willing to pay a price premium for socially responsible products, thus avoiding them being crowded out. These findings are in accordance with our results in treatment Full Info: when consumers are fully aware of the social impact of their purchase decisions, market outcomes reflect their concerns for socially responsible behavior.

A closely related set of papers contains a comparison across market conditions which is similar to the relation between the treatments No Info and Full Info in this paper. Pigors and Rockenbach (2016), in a setting similar to Bartling et al. (2015), also show that once the social dimension becomes known to buyers rather than being private information of the sellers, social responsibility in the market increases. In Feicht et al. (2016), when they face neither commitment nor a possibility that deviating behavior is observed, sellers generate lower donations compared to the case where there is full commitment to donations as promised to buyers at the point of purchase.

Etilé and Teyssier (2016) study the roles of labels in an experimental market. They compare treatments where sellers can acquire labels signifying a minimum amount of donations to a charity to a treatment where no labels can be acquired. They find that only if such labels are credible, i.e., sellers cannot lie, the use of such labels increases social responsibility. Our paper differs from theirs in, at least, two important aspects. First, they cannot say anything about the effectiveness of labels compared to an institutional regime which forces disclosure of the amount of social responsibility. Neither do they allow for sellers to convey information to buyers beyond whether or not a minimum standard is met. Moreover, our design introduces the feature that labels are often complicated and difficult to transmit to consumers, something which is both realistic and relevant for policy makers in so far as markets outside the lab are concerned.^5^

On a broader level, we add to a literature which studies under which conditions market forces are beneficial in generating socially and morally desirable outcomes. Falk and Szech (2013) argue that the presence of markets reduces moral behavior, as indicated by lower prices in markets than in individual decisions for the same externalities (saving the life of a mouse). Yet in a recent paper Bartling et al. (2020a) show that neither Falk and Szech’s data nor their own data support the claim that market interaction erodes moral behavior, but instead it is repeated play that causes the erosion of moral values. Kirchler et al. (2016) also compare individual decision making with a double-auction market and identify the threat of punishment as an effective intervention to increase moral behavior. Sutter et al. (2020) provide evidence that markets with an externality lead to fewer trades, while the effect on prices is ambiguous. The latter two papers use, like us, donations to *unicef* as a proxy for moral behavior. Pigors and Rockenbach (2016) compare monopoly to duopoly markets and show that competition has positive effects on the emergence of more moral behavior. In light of this literature, our design can be interpreted as varying the intensity of competition along the “informativeness-dimension” of the social externality. If there is competition both on the price and the charity dimension, social responsibility increases compared to cases where the latter dimension is not, or only partly, relevant for competitors.

Beyond the literature on social responsibility, our paper also speaks to a literature on information disclosure in markets. Recent evidence (e.g., Jin et al. 2021; Benndorf et al. 2015; Deversi et al. 2021) shows that people have difficulties interpreting non-disclosure as sufficiently negative information, severely limiting the kind of unraveling predicted when agents were sophisticated rather than boundedly rational. Given that in our Choice treatments buyers face a similar task, namely to infer the donation associated with undisclosed offers, it is worth pointing out that if disclosure is sufficiently unambiguous, our results show that buyers behave as if they can infer donations relatively well. An important difference of our paper, also compared to the theoretical literature on disclosure in the presence of boundedly rational consumers (e.g., Ispano and Schwardmann 2018), is that we implement a setting where sellers can choose their donation level, i.e., their product’s quality. Hence, this may be an important driver of the diverging findings, in particular as in most markets quality is endogenous, at least in the medium- to long-run.^6^

## Experimental design

Participants in our experiment interact on markets with four sellers and two buyers. Each participant receives an endowment of $e=20$ [points] in each period. We implement posted-offer markets where buyers arrive sequentially. That is, in each of the 30 periods, one buyer per market is selected at random and faces four offers, one from each seller. This buyer then chooses one (or none) of the four offers and then the second buyer chooses from the remaining offers. A buyer who purchases a product obtains a benefit of $v=100$ and pays a price *p* to the seller. The seller chooses the price $p \in [0,120]$ at which he offers the product, and an amount $d \in \{0,10,20,\ldots ,100\}$ to be transferred to *unicef* in case he sells the product to a buyer. These donations do not influence the buyer’s monetary benefit from the product (*v*), yet higher donations incur higher monetary costs *c*(*d*) for the seller, as shown by the following table:^7^

**Table:** 

*d*	100	90	80	70	60	50	40	30	20	10	0
*c*(*d*)	29	27	25	23	21	18	15	12	8	4	0

Thus, monetary payoffs of buyers and sellers are:

1 $$\begin{aligned}&\Pi ^{\text {b}} = {\left\{ \begin{array}{ll} e \qquad &{} \text {if buyer does not buy} \\ e + v - p \qquad &{} \text {if buyer buys} \end{array}\right. } \end{aligned}$$

2 $$\begin{aligned}&\Pi ^{\text {s}} = {\left\{ \begin{array}{ll} e \qquad &{} \text {if buyer does not buy} \\ e + p - c(d) \qquad &{} \text {if buyer buys} \end{array}\right. } \end{aligned}$$

From a design perspective, implementing the social externality by means of a monetary transfer to *unicef* is attractive for at least two reasons. First, the donation to *unicef* is—as confirmed through a questionnaire at the end of the experiment (see Sect. 5.3)—clearly viewed by the parties trading on the market as a positive externality. Hence, it can suitably capture, in a stylized manner, the choice of a firm how socially responsible the offered product is. Second, money transferred to unicef is not a transfer between market participants, but something that affects people not involved in the transaction, just like many externalities outside of the laboratory, such as environmental effects. Moreover, we view the possibility that participants could, in principle, also donate to *unicef* outside of the experiment as a feature which increases the external validity of the experiment because in many real-world settings consumers and firms can choose between buying more socially responsible products on a market, like a low $\hbox {CO}_{{2}}$ emission vehicle, or can compensate higher emissions through other means.^8^

### Treatments

We implement five treatments which vary the information about the donation. There are two benchmark treatments, one where information on each product’s associated donation is fully disclosed and one where the information is fully hidden: In the No Info treatment, buyers do not observe whether or not donations are made in association with the products offered. The only information buyers have is the price of each product. They also cannot observe this information at a later stage. Neither do sellers observe the donation decisions of the other sellers. In the Full Info treatment, buyers (and the other sellers) observe the donation decisions and levels of all products as well as the products’ prices.

In addition, we run three Choice treatments where information can be disclosed by sellers voluntarily. The three treatments differ in the informational content provided. In treatment Choice-100, whenever sellers decide to reveal the level of donation *d*, customers unambiguously see the production information, i.e., *d* is revealed with probability 100%. In the treatments Choice-85 and Choice-60, the revelation decision of the seller is implemented with probability 85% and 60%, respectively. Thus, when a seller in a given period decides to reveal his donation to the buyers, a random draw by the computer determines whether the decision to reveal and the donation associated with the product are visible to the buyers (and the other sellers) or not. In all three Choice treatments, the decision not to reveal is always implemented with certainty.^9^

### Procedures

The markets in which buyers and sellers interact retain the same composition throughout, that is, there is no re-matching across periods, and participants are aware of this. After all decisions within a round have been made, all participants are informed about their earnings. In addition, throughout the experiment, every subject has access to the full information of his/her past behavior, i.e., prices, earnings and (if known) generated donations. Also, all buyers and sellers are identified through letters and behavior can thus be traced across rounds.^10^

To calculate the total earnings of the participants, the earnings from all periods are added up and converted into Euros at the rate of 130 points = 1 EUR. The same conversion rate applies to donations. These donations are transferred to *unicef* at the day of the experiment. Since donations are both the result of market interaction and sometimes private information (and we wanted to keep this private), we provide all participants with a session average of the donations. This strikes a balance between not revealing much about the individual behavior but at the same time maintaining credibility about our procedures. We furthermore send proof of the donation to all participants (unless they told us they preferred not to receive such an email).

We implemented 12 markets per treatment, leading to 360 participants overall. After the 30 periods of the main experiment, subjects answered a short questionnaire which elicited perceptions about unicef and donation behavior outside the lab (how often the person donated to charities in the past, and how much), as well as demographics such as age, gender and subject of study. The experiments were run at the Cologne Experimental Economics Laboratory (CLER) between May 2017 and October 2018. Recruiting was done via ORSEE (Greiner 2015) and the experiment was programmed in zTree (Fischbacher 2007). Average earnings amounted to 11.64 EUR plus a 4 EUR show-up fee, and average contributions to *unicef* were 68.15 EUR per session.

In this experiment we are powered to detect effects of around one standard deviation (or larger) with 12 markets per treatment condition (MWU-tests, $\alpha =0.05$, power=80%, see Online Appendix A.5 for details). To have sufficient power to be able to judge the (non-)difference between Choice-100 and Full Info (see Result 2), we conducted a replication study in March 2021 consisting of 27 markets each for the treatments Full Info and Choice-100. For a detailed description, including those aspects in which the replication study differs from the initial study, see Online Appendix A.5.

## Predictions

In this section, we derive the predictions for our experiment. We start by considering treatments No Info and Full Info. These two treatments differ only in whether or not buyers can observe the donations chosen by sellers. In treatment No Info, buyers only see the prices sellers ask for. Buyers may believe that some of the higher prices may come from sellers with positive donations. Thus, buyers with social preferences may give up monetary payoff and accept higher prices. Yet, buyers are aware that higher prices are not a reliable signal of positive donations, because they might be an attempt of sellers to achieve higher profits. In contrast, sellers in treatment Full Info compete both on price and the level of social responsibility. A product will be the more attractive to a buyer the lower its price and - for a buyer with some form of social preferences that make her also value money transferred to the charity - the higher the donations associated with a purchase. That donations are always revealed to buyers should lead to a higher level of social responsibility in the market but also to higher prices (because sellers will need to cover the higher production costs) than in No Info.

### Prediction 1

(No Info vs. Full Info) Forcing disclosure of the donation leads to a higher level of social responsibility compared to the case where donations are not visible for buyers. Due to the increased production cost, market prices will be higher when donations are disclosed.

Next, we consider the three Choice treatments. We start by focusing on the Choice-100 treatment, and then move on to Choice-85 and Choice-60. The crucial part of the analysis concerns the beliefs that buyers form when faced with offers that do not have the donation level disclosed. Even though we do not specify a formal model to derive predictions, in the following we sketch a simple yet powerful unraveling intuition which we believe is sufficiently general to apply to our specific setting as well.

Specifically, we posit that a rational buyer holds the belief that the donation level associated with an undisclosed offer equals the average donation level set by all those sellers who do not disclose their donations. Now suppose that among the sellers who choose not to disclose their donation, a share $s<1$ chooses a donation level $d'>0$, while the rest chooses $d=0$. Then, the belief a rational buyer holds about the donation level in undisclosed offers would be $sd'<d'$. But then, a seller who chooses $d'$ is better off disclosing her donation level to separate from those sellers with $d=0$ and thereby increasing the buyers’ willingness to pay for her product. At the same time, if $s=1$, i.e. all sellers who choose not to disclose set $d'>0$ (and buyers believe this to be the case), there is a profitable deviation for a seller to reduce his donation level to $d=0$ because this increases his profits due to the lower production cost without affecting the purchase decision of a buyer (since the donation decision is unobservable). This implies that there should not be any sellers who shroud offers with positive donations.

Importantly, if buyers do not to hold fully rational beliefs, as shown in recent experimental work on disclosure games (e.g., Jin et al. 2021; Benndorf et al. 2015; Deversi et al. 2021), it might be possible that sellers could exploit such biased beliefs by profitably choosing more zero donations compared to Full Info, but not disclosing them to buyers. However, our setting differs from those disclosure games in a number of ways which means that we consider it an open question whether we should expect beliefs to be biased in our case.^11^ We therefore state our prediction based on the assumption that buyers form rational beliefs.

### Prediction 2

(Choice-100 vs. Full Info) Making disclosure of donations voluntary leads to the same market outcome as when sellers are forced to reveal this information. Donations and market prices are identical in treatments Choice-100 and Full Info.

Note, however, that this prediction does not tell us anything about the disclosure rates, i.e., the share of undisclosed offers in treatment Choice-100. Based on the intuition developed above, donations of zero which are disclosed and undisclosed donations should be treated by buyers in a similar manner, which in turn means that for sellers who choose a donation of zero it is irrelevant whether they disclose or not. What we can thus only say is that we do not expect the share of undisclosed offers in treatment Choice-100 to be larger than the share of offers with zero donations in treatment Full Info.

When analyzing the cases where a seller’s decision to reveal the social component of the offer is not implemented with certainty, it is no longer the case that buyers can be sure that an undisclosed donation is due to sellers actively wanting to hide their donation. It could be the case that the seller chooses a high donation but the chance move prevented this donation from being visible to buyers. This means that it is a lot less straightforward for buyers to interpret undisclosed donations in treatments Choice-85 and Choice-60 compared to treatment Choice-100.

Intuitively, we thus predict that such an unreliability in disclosure allows sellers to strategically exploit this more opaque environment. As buyers have to entertain the possibility that an undisclosed donation may in fact also be a high donation, they should, compared to Choice-100 be more inclined to buy products with hidden donations. This, however, should make it a profitable strategy for sellers to (sometimes) choose a low donation and to not disclose. As a countervailing force to this mechanism, however, it also does not seem a profitable strategy for sellers to always choose to not disclose. Since the probability of disclosure is fairly high in both treatments, a seller who decides to disclose a high donation - and this decision actually reaches the buyers - is likely to have a competitive advantage, especially if competing offers have their donation hidden.

Taking these effects together, we predict that the possibilities for sellers to exploit the limited informational value of non-disclosure lead to lower donations compared to the Full Info benchmark. Furthermore, since, as outlined in the previous paragraph, sellers can use the option to disclose strategically in order to exploit the inherent unreliability, we expect that this softens competition between sellers and allows them to charge higher prices, compared to treatment Full Info. We do not make predictions as to whether these effects are larger in treatment Choice-85 or in Choice-60 as this would require a formal model which is beyond the scope of this paper.

### Prediction 3

(Choice-85/Choice-60 vs. Full Info/Choice-100) When donations are voluntary but donation decisions of sellers have limited informational value, donations are lower and prices are higher compared to when there is perfect information.

## Results

We divide the presentation of the results in several parts. First, in Sect. 5.1, we analyze how the treatments compare with respect to donations generated and (in the Choice treatments) the disclosure choices. In Sect. 5.2 we evaluate how market prices and firms’ profits differ across treatments. In Sect. 5.3 we analyze average per period across treatments 5.3 we analyze purchasing behavior in more detail and shed light on the mechanisms behind our results.

Table 1 presents summary statistics of the main variables of interest. The empirical analysis below mainly uses non-parametric tests, e.g., Mann-Whitney-U tests to test for differences across treatments, with market-level averages over the 30 periods as one independent observation.^12^ Since these tests neither allow for taking into account the panel structure of the data, nor controlling for individual-level characteristics (as elicited in our post-experimental questionnaire), we complement the non-parametric analysis with parametric random-effects regressions. The regression tables can be found in the Online Appendix; we mention in the text those cases where the results from the latter are noteworthy.

**Table 1:** Summary statistics of main variables of interest

	Full Info	Choice-100	Choice-85	Choice-60	No Info
% of buyers who bought	99.6 (0.8)	99.7 (0.6)	100 (0)	100 (0)	99.7 (1.0)
Donations (offered)	61.7 (23.3)	44.5 (26.7)	31.9 (13.5)	42.9 (19.0)	15.0 (13.1)
Donations (sold)	62.1 (27.2)	43.6 (30.0)	27.3 (16.4)	40.1 (19.9)	11.5 (12.5)
% revealed (intention)	100	74.7 (12.6)	63.9 (18.7)	65.6 (15.0)	0
% revealed (realized)	100	74.7 (12.6)	55.6 (16.4)	39.2 (7.5)	0
% revealed (intention, sold only)	100	75.6 (16.6)	59.7 (23.4)	61.9 (18.3)	0
% revealed (realized, sold only)	100	75.6 (16.6)	56.3 (22.5)	48.1 (13.2)	0
Prices (offered)	43.9 (5.7)	41.7 (12.8)	39.5 (8.1)	47.1 (9.8)	43.1 (8.1)
Prices (sold)	40.6 (6.4)	36.3 (13.7)	34.4 (9.7)	44.0 (10.7)	39.5 (8.9)
Payoff buyer	79.1 (6.3)	83.5 (13.6)	85.6 (9.7)	76.0 (10.7)	80.4 (8.9)
Payoff seller	30.8 (2.8)	31.3 (5.1)	32.7 (4.4)	35.7 (3.6)	37.7 (3.9)

The table reports market averages and standard deviations (in brackets) for the different treatments

### Donations

#### Full Info vs. No Info

Whether or not donations are visible has a drastic influence on market outcomes. In line with Prediction 1, donations generated by a purchase in Full Info are almost five times larger than those in No Info: When donations are always observed by buyers, their purchase decisions generate donations of 62.1 points, compared to 11.5 points when donation levels are private information of an individual seller ($p<0.001$, MWU-test). Table A1 in the Online Appendix shows that the corresponding treatment effect, when we control for individual buyer characteristics, is estimated as -55.7 ($p<0.001$).

**Fig. 1:**
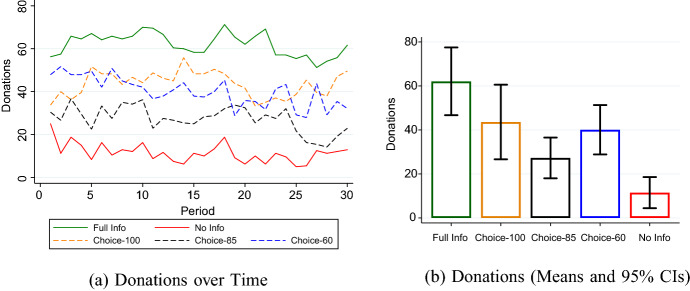
Social Responsibility in the Market. (**a**) plots average donations per period over the thirty periods of the experiment for each of the five treatments. (**b**) shows means and 95% confidence intervals of the donations generated on average per period across treatments

The difference between the two treatments is especially pronounced at both extremes of the possible donation levels (see Figure A1 in the Online Appendix). In No Info, 62.0% of products bought do not generate any donation, but this is only true for 16.9% in Full Info. The highest donation of 100, however, is realized in only 4.2% of purchases in No Info, compared to 44.2% in Full Info. Remarkably, there is no time trend in donations as the experiment progresses (see Fig. 1a).^13^

##### Result 1

When the social dimension of products is visible to buyers (Full Info), donations are almost five times larger than when buyers do not observe it (No Info). This confirms Prediction 1.

#### Choice-100 vs. Full Info

Average donations in Choice-100 are much higher than in treatment No Info, but appear to be smaller than in Full Info (see Fig. 1b). In light of our predictions, especially the latter comparison is of interest. On average, in Choice-100, generated donations amount to 43.6 points, 29.8% less than in Full Info. Based on a MWU-test, this difference is however not statistically significant ($p=0.166$). When considering the parametric alternative, we do estimate a significant treatment difference of −18.7 ($p=0.044$, see Table A1 in the Online Appendix).

Mirroring the comparison between No Info and Full Info, the difference, if any, between the two treatments is largely driven by behavior at the extremes of the possible donation schedule. Compared to Full Info, the share of accepted offers with zero donations is 7.8 percentage points higher (16.9% vs. 24.7%), whereas the share of accepted offers with the maximum donation level of 100 is 17.6 percentage points lower (44.2% vs. 26.6%).

Hence, while donations seem to be somewhat affected by making disclosure voluntary, we fail to establish unambiguous statistical significance. In order to investigate whether this is due to a potential lack of power to detect a small but significant effect of voluntary disclosure on donations, we conduct a replication of Full Info and Choice-100 with 27 markets per treatment condition (for full details of the replication, see Online Appendix A.5). In this replication average donations in the two treatments turn out to be very similar and we find no significant difference between the two conditions ($p=0.736$, MWU-test). Taking these findings together, we can state the following result:

##### Result 2

When sellers can choose whether to reveal the social impact of their products to buyers and disclosure is perfect (Choice-100), donations are not significantly different compared to the case where sellers are forced to do so (Full Info). This supports Prediction 2.

#### Imperfect disclosure (choice-85 and choice-60)

Prediction 3 tells us that when sellers decide to inform consumers about their level of donations but disclosure is imperfect, this should lower donations, compared to the Full Info situation. Our results indicate that this is indeed the case. Donations amount to an average of 27.3 per sold product in Choice-85, while in Choice-60, the average is 40.1. In both cases, this constitutes a significant decrease relative to donations in Full Info ($p=0.003$ and $p=0.0496$, MWU-test, respectively). Consistent with this result, the corresponding treatment effects are given by -38.0 ($p<0.001$, Choice-85) and -27.0 ($p=0.001$, Choice-60).

We can furthermore see a similar bimodality in donations as in the other treatments. In Choice-85, 11.9% of realized donations yield the highest donation, while 39.9% of accepted offers do not generate any donation at all. These shares are about halfway between No Info and Choice-100. For Choice-60, the share of realized donations of zero is at 36.0% and therefore comparable to behavior in Choice-85. Perhaps surprisingly, the share of maximal donations is substantially higher, at 23.3%. We discuss possible reasons for this in Sect. 5.3 below.

##### Result 3

When sellers can choose to reveal the social impact of their product, but the informational content of the choice to disclose is limited, donations decrease by 56.1% in treatment Choice-85 and by 35.5% in treatment Choice-60, compared to when sellers are forced to do so and there is full transparency (Full Info). This supports Prediction 3.

Comparing donations in Choice-100 and in the two imperfect disclosure treatments we do not establish a significant difference when using the non-parametric test. Yet, the regression approach in Table A1 in the Online Appendix indicates a significant treatment effect of -19.3 ($p=0.017$) for the comparison of Choice-100 to Choice-85, but no significant difference for the comparison of Choice-100 to Choice-60 ($p=0.985$).^14^ Taken together, we do not find unambiguous evidence for an effect between Choice-100 and the other choice treatments. Hence, uncertainty remains about how sensitive donations are to a change in disclosure probability. We will discuss this issue further in Sect. 5.3.

#### Disclosure choice

Next, we analyze the disclosure behavior of sellers. Across the three Choice treatments, the share of offers where a seller decided to disclose the donation is 69.0%. The share of offers where a seller decided to disclose the donation is slightly higher in Choice-100 (74.7%) compared to the other two (63.9% in Choice-85 and 65.6% in Choice-60, respectively), though this difference is not statistically significant. Of course, in these two treatments, the intentions of the sellers do not correspond to the share of disclosed donations in the market. In Choice-85, 55.6% of offers feature a visible donation, for Choice-60 this number drops to 39.2%.

As discussed in Sect. 4, we would expect sellers to only hide their donation if they offer a donation of zero. This is indeed the case in our data. Only 9.1% of offers hide a positive donation level. Conversely, of those offers with a donation level of zero, for only 5.4% of them did the seller intend to reveal the donation to buyers. Hence, sellers, by and large, use the possibility to hide the social impact of their product when this product is socially irresponsible.

### Market prices and profits

**Fig. 2:**
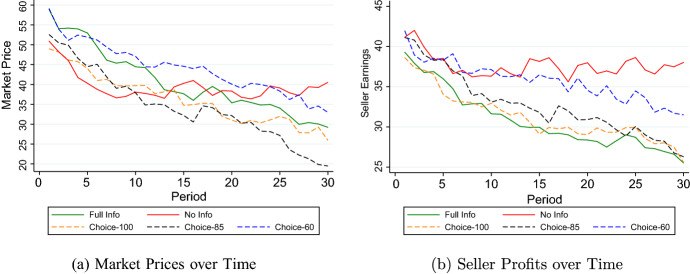
Prices and Profits in the Market. (**a**) plots average market prices, i.e., accepted offers, per period over the thirty periods of the experiment for each of the five treatments. (**b**) plots average seller earnings per period over the thirty periods of the experiment for each of the five treatments

#### Full info vs. no info

How do these considerable differences in donations translate into market prices? Notice that buyers almost always buy a product (99.6% of cases in Full Info and 99.7% of cases in No Info). Remarkably, however, there is no significant difference in market prices between the two treatments. While in treatment Full Info a product is sold at an average price of 40.6, in No Info, buyers pay on average 39.5 ($p=0.564$, MWU-test). In treatment No Info, however, market prices decline only in the first few periods while afterwards they remain almost constant over the 30 periods. In contrast, in treatment Full Info we observe a significant decrease in market prices by −0.902 points per period ($p<0.001$), see Fig. 2a.^15^

Since sellers in Full Info sell products which have a higher production cost due to the higher donations, they obtain lower profits than sellers in No Info: Sellers in Full Info earn on average 30.8 points compared to 37.7 points in No Info ($p<0.001$, MWU-test). Controlling for observables, the corresponding treatment effect amounts to 7.2 points ($p<0.001$), see Table A1 in the Online Appendix. Thus, the higher production cost due to the higher donations in Full Info, is almost fully borne by the sellers. In line with the result on price dynamics reported above, seller profits decrease over time in treatment Full Info while in No Info they do not.^16^

We note that this is not what we should observe based on Prediction 1. As discussed above, we would have expected prices to be lower in No Info since production cost are lower as well. The fact that prices are similar and follow a different time trend indicates that there are differences in the intensity of competition between the two treatments. We establish in Sect. 5.3 that purchase decisions by buyers in No Info hamper effective competition between sellers.

##### Result 4

Market prices are not significantly different between No Info and Full Info. Sellers thus earn higher profits in treatment No Info. Both findings are at odds with Prediction 1.

#### choice-100 vs. full info

Average market prices in Choice-100 are 4.3 points lower than in Full Info. This difference is not statistically significant when using matching group averages (36.3 vs. 40.6, $p=0.488$, MWU-test). The same result of a non-significant decrease is established when using the parametric approach (treatment effect of -4.62, $p=0.184$). In both treatments, prices decline over the course of the experiment (see also Table A1 in the Online Appendix).^17^

As discussed in the previous subsection, hidden donations overwhelmingly mask donations of zero. An important question is whether this is reflected in the market prices. In particular, we might gain some insights as to whether buyers understand the strategic incentives of the sellers and hold unbiased beliefs. Given the evidence on limited strategic thinking in disclosure games (e.g., Jin et al. 2021; Benndorf et al. 2015; Deversi et al. 2021), it would be possible that buyers make wrong inferences about what undisclosed donations in Choice-100 imply. Then, they may mistakenly pay higher prices than they would for offers where they know that donations are zero.

However, our results show that in our setting buyers hold relatively accurate beliefs, correctly predicting that hidden donations mask offers with zero, or at best very small donations. This can be inferred from the fact that in Full Info, the average price paid for a good with a donation of zero is 26.4, compared to an average of 28.3 which is paid for a good with its donation hidden ($p=0.681$, t-test with clustered s.e.).

Turning to seller profits, we find that there is no meaningful difference between Full Info and Choice-100. Sellers in Full Info earn 30.8 points on average, compared to 31.3 points in Choice-100. This difference is not significant ($p=0.908$, MWU-test). Also, in both treatments profits decline over time at a similar rate (−0.392 points per period in Full Info and -0.331 per period in Choice-100, $p=0.397$, see Table A1 in the Online Appendix). Hence, while the decrease in donations reduces the production cost for the sellers, the fact that the prices they charge are lower, especially when the donations are not revealed means that endogenizing the disclosure choice does not affect seller profits.

##### Result 5

Market prices are similar between Choice-100 and Full Info. Seller profits do not differ between the two treatments. This supports Prediction 2.

#### Imperfect disclosure (choice-85 and choice-60)

Prediction 3 states that when the informational content of disclosure is limited, prices are predicted to be higher compared to when there is full information or perfect disclosure. Our results are at odds with this prediction. The average price paid in Choice-85 is 34.4 which is significantly lower than the price paid in Full Info, 40.6 ($p=0.043$, MWU-test). The price paid in Choice-60, however, is significantly larger than the price in Choice-85 (44.0, $p=0.043$, MWU-test), consistent with the intuition that lower donations lead to lower prices. Hence, there seems to be an important difference between the two treatments with respect to whether competition in the market drives down prices or not. We will return to this result further below, in particular when discussing it in relation to the differences in prices between No Info and Full Info as we argue that similar forces may be at play in both cases.

Turning to seller earnings, we find that while prices are significantly different between the two treatments, for profits this is not the case. While profits in Choice-60 are 3.0 points higher than in Choice-85, this difference fails to reach statistical significance ($p=0.119$, MWU-test). This is explained by the slightly higher donations in Choice-60. Looking at the dynamics, we observe that seller earnings in both treatments decline over time, but the decrease per period is significantly stronger in Choice-85 than in Choice-60 (-0.431 vs. -0.264, $p=0.043$, see Table A1 in the Online Appendix). Moreover, profits in Choice-60 are higher than profits in both Choice-100 (35.7 vs. 31.3, $p=0.043$, MWU-test) and Full Info (35.7 vs. 30.8, $p=0.004$, MWU-test) while there is neither a difference between profits in Choice-85 and profits in Choice-100 (32.7 vs. 31.3, $p=0.564$, MWU-test) and Full Info (32.7 vs. 30.8, $p=0.225$, MWU-test).

##### Result 6

Market prices in Choice-85 are lower than prices in Full Info. Prices in Choice-60 are, however, almost 10 points higher than in Choice-85 and not significantly different from prices in Full Info. This is at odds with Prediction 3. Seller profits increase when the informational content of disclosure is sufficiently limited (Choice-60).

Comparing profits across all five treatments, it is noteworthy that they can be ranked according to their informational content. In particular, seller profits are highest in No Info, followed by Choice-60 and Choice-85, which, in turn, slightly, but not significantly, outperforms the other two cases where disclosure leads to perfect information about the level of social responsibility associated with the product.^18^

### Discussion of the mechanisms

The results from the previous two subsections provide evidence that the disclosure (and its informativeness) of the social responsibility of products plays a crucial role both for the amount of social responsibility obtained in the market as well as for the profitability of firms competing. The goal of this subsection is to analyze the mechanisms behind the results and to highlight the implications for policy interventions, such as regulation. The discussion is guided by four questions opened up by the results.

#### Question 1

Why are seller profits in No Info higher than in Full Info?

Our two treatments where disclosure of social responsibility is exogenously determined reveal a meaningful difference both in the degree of social responsibility in the market as well as in seller profits. While the first is in line with the hypothesis, and indeed previous evidence (e.g., Pigors and Rockenbach 2016; Etilé and Teyssier 2016), the fact that sellers actually benefit from a regime where social responsibility is not revealed to consumers warrants closer scrutiny. In particular, while the externality is hidden from consumers, it is not absent from the market and buyers are aware that sellers had to make a decision how socially responsible to behave. Thus, buyers could try to infer donations from the only information they have, i.e., the prices posted by the sellers, but this exercise may well prove futile if sellers anticipate such behavior.

Strikingly, we find that in 30.6% of decisions, a buyer does not buy from the cheapest seller. Instead, she seems to deliberately favor a seller with a higher price, suggesting that she expects this seller to have chosen a higher donation. While it is indeed the case that in No Info higher prices do signal higher donations (an increase in donations by one (10 point) increment implies a 2.6-point increase in the price, $p<0.001$, see Table A3 in the Online Appendix), buyers are far from perfect in inferring donations from prices (26.7% of choices are dominated by an offer with a lower price and a higher, but unobserved, donation). The fact that in Full Info only in 7.3% of cases buyers choose offers which are dominated by another offer with both a lower price and a higher observed donation, indicates that choosing offers with higher prices in No Info is not caused by erroneous behavior but stems from conscious decisions of buyers.^19^

It remains speculative whether buyers in No Info sometimes buy higher-priced products because they firmly believe that they are able to correctly predict undisclosed donations, or whether other types of reasoning, such as motivated beliefs (i.e., convincing oneself that paying a higher price is the more socially-responsible action) play an important role as well. Yet, it is clear that this buyer behavior has consequences for the competitiveness of the market: that in No Info in almost one third of the cases a seller can sell his product although it is not the cheapest (while dominated choices are rare in Full Info) yields a reduced intensity of competition in No Info. This explains why market prices in No Info display no decreasing trend, while in Full Info the steady decline of market prices suggests that sellers actively try to undercut each other.

At the end of the experiment, we elicited how positively subjects evaluate the charity which receives the donations. Based on these answers, we use a binary measure to classify subjects as either having a positive or a neutral/negative view.^20^ In Full Info, buyers who hold a positive view about the charity generate almost three times more average donations than those who do not (74.0 vs. 26.3, $p=0.001$, MWU-test on individual buyer averages). At the same time, in treatment No Info, those who think positively about the charity buy a higher priced product more than five times more often than those who do not (40.0% vs 7.6%, $p=0.016$, MWU-test on individual buyer averages).^21^ Hence, the willingness to pay a higher price under the belief that this may generate positive donations is positively correlated with a favorable perception of the social externality.

Interpreting the high prevalence of choosing offers with a higher price in light of the question of (un)informative labeling and its effect on market outcomes, we predict that if there are no possibilities for sellers to inform consumers about the social responsibility of their products, e.g., because this dimension is too complex to convey, this has additional anti-competitive effects. Since consumers would have to make inferences about product characteristics that they care about but have no way of finding out, they mistakenly infer too much from prices, hampering competition and leading to higher prices.

#### Question 2

How does limited transparency affect donations when disclosure is voluntary?

Our findings that donations in Choice-100 come close to Full Info (Result 2) and that donations in Choice-85 and Choice-60 decrease compared to Full Info (Result 3) suggest that the decrease in donations is not caused by the fact that disclosure is voluntary, but by the limited informational content of disclosure. However, as described above, our data remains inconclusive when comparing donations in Choice-85 and Choice-60 to donations in Choice-100. In particular, we cannot say by how much the observed decline in social responsibility between Full Info and Choice-60 and Choice-85, respectively, can be attributed to the effect of making disclosure voluntary and by how much to the additional effect of a decrease in the reliability of information. One reason for this is that our initial data, as becomes clear when looking at Fig. 1b again, features rather large confidence intervals, especially in Choice-100. Therefore, in order to shed more light on whether donations in Choice-100 are indeed different from Full Info - which would indicate that it is voluntary disclosure per se which decreases donations - we conducted a high powered replication of treatments Choice-100 and Full Info. As already described in Sect.5.1 above, this replication shows that the effect of just making disclosure voluntary is small.

This observation is corroborated when estimating a random-effects regression of realized donations on a set of treatment dummy variables including all observations from the initial experiment and the replication. Since the replication was conducted in a somewhat different setting than the main experiment, we include a dummy variable indicating data coming from the replication study.^22^ As shown in Table A11, Column (1) in the Online Appendix, we estimate a small and insignificant effect ($-3.6$ points, $p=0.530$) of making disclosure voluntary on donation levels between Full Info and Choice-100. The effect of reducing the disclosure probability from 100% (Choice-100) to 85% (Choice-85) is estimated to cause a reduction in donations of 26.5 points ($p<0.001$). The effect of moving from 100% (Choice-100) to 60% (Choice-60) is estimated to decrease donations by 16.0 points ($p=0.039$).

Hence, while we acknowledge that some uncertainties remain regarding the interpretation of the findings of the three Choice treatments, we view these results as suggestive evidence that the observed decline in social responsibility between Full Info and Choice-60 and Choice-85, respectively, is to a large extent due to the effect of a decrease in the reliability of information, while simply making disclosure voluntary does not affect social responsibility much.

#### Question 3

What differences exist between Choice-85 and Choice-60 and why?

A more detailed analysis of the two imperfect disclosure treatments can provide further insights into how conditions which make it hard for firms to provide easily-interpretable and unambiguous information about the social responsibility of their products to consumers, affect market outcomes. As discussed above, prices in Choice-60 are significantly higher compared to Choice-85 (Result 6) and sellers earn significantly more in Choice-60 than in treatments Choice-100 and Full Info. Seller earnings in Choice-85, however, are not significantly different from those in Full Info and Choice-100, respectively

To make sense of these results, note that given the disclosure decisions of the sellers and the imperfect implementation probability of 60%, only for 39% of offers in Choice-60 do consumers know the donation level. In these cases, the decision which product to buy bears some resemblance to the No Info treatment in the sense that buyers might engage in inferences about the donation level based on the prices. Corroborating our earlier finding for the No Info treatment, we now find that in 13.8% of decisions in Choice-60, a buyer buys a product with a hidden donation which is not the cheapest among those with a hidden donation. In comparison, in Choice-85 this only happens in 4.3% of cases.

This suggests that behavior in Choice-60 is similar to No Info in the sense that buyers try to predict donation levels for those donations they do not observe. The difference between the two treatments is that in Choice-60 buyers do sometimes get information about seller behavior. This might allow sellers to create a reputation for being a socially responsible seller (remember that sellers’ identities are known to buyers and do not change over time). This may explain why there are relatively many donations of 100 in this treatment, compared to Choice-85 (23.3% vs. 11.9%). At the same time, having developed a reputation for being socially responsible could create leeway for sellers to charge higher prices, consistent with what we observe in the data. We discuss the role that reputation plays in the Choice treatments in more detail below.

#### Question 4

What role does reputation play in the Choice-treatments?

In the three Choice-treatments a buyer will regularly be confronted with offers with hidden donations. But, since sellers are identified through letters A to D in the experiment, buyers have the possibility to track seller behavior across periods. This might allow them to make inferences about hidden donations based on sellers’ past behavior. At the same time, sellers might therefore be able to develop a reputation for being, for example, a socially responsible seller. Buyers might then be more likely to also buy from such a seller even if the donation is hidden in some of the periods.

In order to analyze whether reputation plays a role in our markets, we estimate, similar to Bartling et al. (2015), a conditional logit choice model. We assume that a buyer’s utility from buying a product for a price *p* and a donation *d* is given by $u = \beta (120 - p) + \theta ^b d$. Here, $\beta >0$ denotes the weight a consumer puts on her own monetary payoff, while $\theta ^b$ captures the extent to which a consumer cares about the level of social responsibility of a product, i.e., the donations generated by purchasing it. When estimating the coefficients $\beta$ and $\theta ^b$ we treat donations which are observable to the buyer differently from those which are not and capture the latter via a dummy variable equal to one whenever a donation is not visible to the buyer.

Our main interest, however, lies in using this conditional choice model for quantifying a potential effect of reputation. To do that, we include an additional variable into this model which captures how often a buyer previously bought from a specific seller. More precisely, for each alternative available to a buyer we calculate the share of offers she accepted from this specific seller in the past. Hence, controlling for the price and donation of a given offer, we check whether a buyer is more likely to buy from a seller he already bought more from in the past.

Table 2 presents the results from estimating such a model. We first note that we estimate, as should be expected, a positive value for $\beta$ as well as $\theta ^b$, with the ratio of the two being between five and seven, depending on the specification, indicating that buyers are willing to give up one point of their own monetary payoff to increase donations by five to seven points. We then note the share of offers previously bought from this seller has a positive effect on the utility associated with a specific alternative (see columns (1) and (2) in Table 2). When allowing for this effect to vary across treatments, we find a significant effect only in the Choice-60 treatment. To quantify this effect, a ten percentage points increase in the share of offers bought from a seller is comparable in magnitude to a price increase of about one point. In Choice-85 and Choice-100, the effect is not significant.

**Table 2:** Conditional logit choice model for choice-treatments

	(1)	(2)	(3)	(4)
Weight on Buyer Earnings ($\beta$)	0.133***	0.143***	0.136***	0.146***
	(0.020)	(0.021)	(0.020)	(0.021)
Weight on Donations ($\theta ^b$)	0.019***	0.022***	0.028***	0.029***
	(0.003)	(0.003)	(0.009)	(0.008)
Donations ($\theta ^b$) $\times$ Choice-85			−0.012	−0.012
			(0.010)	(0.010)
Donations ($\theta ^b$) $\times$ Choice-60			−0.010	−0.008
			(0.009)	(0.009)
Hidden Donation Dummy	−0.133	−0.081	0.060	−0.027
	(0.157)	(0.163)	(0.280)	(0.277)
Hidden Donation Dummy $\times$ Choice-85			−0.283	−0.063
			(0.347)	(0.338)
Hidden Donation Dummy $\times$ Choice-60			−0.350	−0.196
			(0.387)	(0.406)
Seller Share	0.629***	0.470**	0.159	0.059
	(0.221)	(0.213)	(0.216)	(0.247)
Seller Share $\times$ Choice-85			0.311	0.349
			(0.404)	(0.518)
Seller Share $\times$ Choice-60			1.036**	0.814*
			(0.456)	(0.441)
Case-specific individual variables	No	Yes	No	Yes
Observations	7302	7302	7302	7302
Cases	2086	2086	2086	2086
Cluster	36	36	36	36

The table shows estimates from Mc Fadden’s conditional logit choice models. We assume that a buyer’s utility from buying a product for a price *p* and a donation *d* is given by $u = \beta (120 - p) + \theta ^b d$. $\beta >0$ denotes the weight a consumer puts on her own earnings, while $\theta ^b$ captures the extent to which a consumer cares about the donations to unicef. The seller share indicates the share of offers a buyer accepted from the respective seller in the past. All models contain data from Choice-100, Choice-85 and Choice-60, with Choice-100 serving as the reference category. The treatment variables are binary. Columns (2) and (4) also contain case specific individual variables including period-specific dummy variables and characteristics of the buyer. Standard errors (clustered at the market level) in parentheses.* $p<0.10$, ** $p<0.05$, *** $p<0.01$

Hence, corroborating our results on the dominated choices presented above, we do indeed find evidence that some form of reputation plays a role in those Choice-treatments where disclosure is imperfect. However, we note that our experiment was not designed to specifically analyze the role of reputation. It would be an interesting avenue for further research to analyze the role of reputation more thoroughly.

## Implications and concluding remarks

In this paper, we have analyzed the role of social responsibility (SR) in markets. We show that disclosure of SR information has large and positive effects compared to a regime where producers are unable to disclose the social responsibility of their products to consumers (No Info). We find that when voluntary disclosure provides unambiguous information for customers (Choice-100), the level of social responsibility comes close to the outcome under full and externally enforced disclosure (Full Info). Yet, in the cases where producers cannot be sure that the provided SR information has full informational content for consumers (Choice-85 and Choice-60), SR in production is significantly lower, compared to Full Info. Yet, since our data does not provide an unambiguous statistical difference between the three choice treatments, the sensitivity of social responsibility to changes in the disclosure probability cannot be precisely determined.

We believe that for policy makers and regulators interested in influencing the level of SR in the market, several implications of our results are noteworthy. First, we have established that the more opaque the disclosure of SR attributes, the larger the firms’ profits. Hence, one should expect firms to oppose, on the one hand, those kinds of regulations which enforce disclosure of such attributes, but also, on the other hand, initiatives which facilitate comparisons of products along this dimension.

On a more positive note, our results show that the benefits from full transparency and comparability can be large compared to a situation with no information, even if one relies on voluntary disclosure by firms. Especially as too heavy-handed regulation may be difficult to implement and enforce, voluntary disclosure can go a long way. Our results indicate that competition on the SR dimension is sufficiently strong to deliver both high disclosure rates as well as high levels of SR.

Importantly, however, our results indicate that the above effects of voluntary disclosure are limited in markets where the producer faces uncertainty about whether disclosed SR information reaches the consumers. We studied a situation in which the SR of the product - when communicated to consumers - was unambiguously clear. The overwhelming majority of subjects knew *unicef* and rated it highly positive. Yet it seems reasonable to assume that the effects of voluntary disclosure get weaker when SR information is less meaningful, either because the provided information captures only a part of a complex and opaque production process or a multiplicity of labels distorts the informational content and limits comparability. These results thus provide empirical support for theoretical models which posit that consumer confusion about the informational content of labels decrease the incentive for firms to disclose these attributes (e.g., Harbaugh et al. 2011). They also suggest that an important role for policy makers lies in making it easier for firms to communicate to consumers how to assess the SR of products, for example through reliable and easy-to-interpret information.

## Supplementary Information

Below is the link to the electronic supplementary material.

Supplementary file1 (PDF 889 kb)

Supplementary file1 (PDF 289 kb)

## Footnotes

1 Taking the market for organic foods as one example, in the United States, the share of organic foods compared to total food sales in 2017 is estimated to be at 5.5% (OTA , 2018). In Europe, Denmark has the highest share of organic retails sales at 13.3% of the total market (FiBL, 2017).

2 See, for example, Bartling et al. (2015), Bartling et al. (2019), Bartling et al. (2020) or Pigors and Rockenbach, (2016) for laboratory evidence and Hainmueller et al. (2015) or Elfenbein and McManus (2010) for field evidence.

3 For instance, for the United States, the magazine *Consumer Reports* presents a detailed guide navigating through the different fair trade labels for coffee, identifying five trustworthy ones and, in addition, warning consumers about a number of meaningless claims. (Dabney, 2017). The German Federation for the Environment and Nature Conservation (BUND) lists seventeen different labels that are currently used to certify ecological and sustainable standards in production (BUND, 2019).

4 Using transfers to *unicef* as a proxy for SR has been done previously, e.g., in Sutter et al. (2020) and Kirchler et al. (2016). We justify our choice of using this proxy for SR further in Sect. 3 when presenting the details of our experimental design

5 The paper by Engelmann et al. (2018) addresses the argument of external validity of experimental studies of socially responsible behavior in markets. Reassuringly, they demonstrate that buyers who show high levels of social responsibility in experimental markets, also have higher willingness-to-pay for real fair trade chocolate over conventional one.

6 A further, though somewhat more distant connection can be drawn from our work to the literature on experimental “lemon markets” where quality choice is endogenous (e.g., Holt and Sherman, 1990; Henze et al., 2015). This is because if one interprets SR production as the quality of a product, our No Info and Full Info treatments can be understood as capturing similar ideas as in studying markets with full or asymmetric information on quality. Our finding that social responsibility is lower in No Info than in Full Info is consistent with the emergence of a “lemons outcome” found in this literature.

7 This cost function exhibits decreasing average cost which can be seen as capturing some implicit fixed cost in socially responsible production. For the sake of experimental simplicity, we did not implement fixed costs explicitly.

8 Due to the fact that a donation *d* has a cost of $c(d) < d$ for all *d* for a seller, it is however more efficient for market participants as a whole to generate donations in the experiment rather than outside of the experiment. This is not necessary for the external validity of the design, but makes it less attractive for participants to substitute generating donations in the experiment by donating afterwards outside of the experiment.

9 As a robustness check we also ran a variant of treatment Choice-100 where we introduce a disclosure cost of 3 but at the same time shift the cost *c*(*d*) down by 3 points. While the cost associated with disclosure in this treatment are identical to Choice-100, this is not the case for sellers who decide not to disclose. Hence, buyers could be more likely to believe that sellers do not disclose positive donations in order to save on the disclosure cost. In Section A.6 in the Online Appendix we show that behavior is similar to Choice-100 and discuss the implications of this result. We refrain from including it in the main text because due to the different disclosure costs, this treatment is not directly comparable to the rest.

10 For a (translated) version of the experimental instructions, see Online Appendix B.

11 The most important difference is the fact that we consider a setting in which the informed party (sellers), selects the relevant quantity (donation) before deciding whether to disclose it or not. In the disclosure games cited above, the disclosure choice typically concerns a quantity which has been determined randomly.

12 The complete set of all pairwise tests for the main outcome variables can be found in Online Appendix A.3.

13 See Table A1 in the Online Appendix for the regression that formally establishes this result by allowing for a time trend in donations.

14 Also, if we - based on Result 2 - pool Full Info with Choice-100 and Choice-85 with Choice-60, respectively, we can test for a difference in donations between these two pooled samples which provides a high powered test of Prediction 3. When doing so, we find a significant difference (52.8 vs. 33.7, $p=0.036$, MWU-test).

15 In No Info, we estimate the average per-period decrease to be −0.184 ($p=0.222$). In both case we use RE-regression of prices as the dependent variable on a linear time trend. See Table A1 in the Online Appendix.

16 In particular, Table A1 in the Online Appendix shows that seller profits in Full Info decrease by 0.392 points per period ($p<0.001$) while in No Info we estimate no significant time trend (-0.058 per period, $p=0.409$).

17 These results are confirmed in the replication study we conducted for treatments Full Info and Choice-100, see Online Appendix A.5 for details.

18 The difference in profits between No Info and the other treatments is significant at the 5%-level for all treatments but Choice-60. Table A1 in the Online Appendix provides the treatment effects from the random-effects regression and confirms the findings presented in this section.

19 The full analysis of dominated choices in the different treatments can be found in Table A4 in the Online Appendix.

20 The answers were given on a scale from 1 (which corresponds to considering the charity as “very worthy of support”) to 7 (corresponding to “not at all”). We classify subjects as having a positive view of the charity if they answer either “1” or “2”. Across all participants in our sample, about 35% of participants answer “1” and another 35% answer “2”, which is why we chose this classification. There is no significant difference in the view of the charity across treatments; they are fully balanced also when comparing buyers and sellers separately.

21 These results are robust to using “3” or “4” as a cutoff and also when using the measure as a dependent variable in a regression framework: In Full Info a higher value of the “charity variable” (i.e., a more negative view of the charity) has a significantly negative effect on average donations; in No Info a higher value significantly reduces the probability to choose a higher priced product.

22 As described in more detail in Online Appendix A.5, the main (and unavoidable) difference was that due to the COVID-19 pandemic, the replication study had to be conducted online.
