# Supplementary material for: Revealing good deeds: disclosure of social responsibility in competitive markets
Source: Exp Econ. 2022 Mar 23;25(5):1349–73. doi: 10.1007/s10683-022-09752-z (PMC8940984; doi:10.1007/s10683-022-09752-z)
Supplement: Supplementary file 2 — Supplementary file1 (PDF 289 kb) [file 10683_2022_9752_MOESM2_ESM.pdf]

Online Appendix

for the paper

"Revealing Good Deeds: Disclosure of Social Responsibility in Competitive Markets"

by Sören Harrs, Bettina Rockenbach and Lukas M. Wenner

accepted for publication at Experimental Economics

A Additional Analyses

A.1 Regressions

	Donations		Price		Seller Earnings	
	(1)	(2)	(3)	(4)	(5)	(6)
Choice-100	-18.735**	-21.661**	-4.615	-7.666	0.507	-0.441
	(9.311)	(10.553)	(3.475)	(5.168)	(1.586)	(2.320)
Choice-85	-37.987***	-36.004***	-7.906**	-6.409*	1.960	2.557
	(8.070)	(9.583)	(3.276)	(3.821)	(1.457)	(2.113)
Choice-60	-27.023***	-21.855**	1.831	-1.151	4.826***	2.844*
	(8.127)	(8.970)	(3.179)	(3.529)	(1.282)	(1.648)
No Info	-55.719***	-56.198***	-2.436	-13.546***	7.148***	1.970
	(7.936)	(8.136)	(3.253)	(3.761)	(1.389)	(1.868)
Period		-0.252		-0.902***		-0.392***
		(0.239)		(0.120)		(0.044)
Choice-100 × Period		0.189		0.198		0.061
		(0.496)		(0.217)		(0.072)
Choice-85 × Period		-0.128		-0.096		-0.039
		(0.425)		(0.183)		(0.081)
Choice-60 × Period		-0.333		0.193		0.128**
		(0.302)		(0.150)		(0.063)
No Info × Period		0.031		0.718***		0.334***
		(0.247)		(0.193)		(0.082)
Observations	3600	3600	3593	3593	7200	7200
Cluster	60	60	60	60	60	60

Notes: The table shows the results from random-effects regressions on dummy variables indicating the treatment and the individual-specific control variables described in Section A.1.1. Columns (1) (3) and (5) also contain Period-specific dummy variables. Standard errors are clustered by market. * $p < 0.10$, ** $p < 0.05$, *** $p < 0.01$.

Table A1: Treatment Effects for Main Outcomes

	Offered Donations		Buyer Earnings	
	(1)	(2)	(3)	(4)
Choice-100	-16.395*	-20.202**	4.586	7.788
	(8.850)	(9.035)	(3.445)	(5.131)
Choice-85	-29.835***	-27.619***	8.079**	6.607*
	(7.822)	(7.639)	(3.258)	(3.747)
Choice-60	-19.021**	-12.284	-1.703	1.307
	(8.297)	(7.878)	(3.169)	(3.473)
No Info	-46.812***	-46.621***	2.363	13.669***
	(7.205)	(6.070)	(3.282)	(3.685)
Period		-0.021		0.903***
		(0.202)		(0.121)
Choice-100 × Period		0.246		-0.207
		(0.395)		(0.220)
Choice-85 × Period		-0.143		0.095
		(0.350)		(0.184)
Choice-60 × Period		-0.435*		-0.194
		(0.264)		(0.151)
No Info × Period		-0.012		-0.729***
		(0.213)		(0.197)
Observations	7200	7200	3600	3600
Cluster	60	60	60	60

Notes: The table shows the results from random-effects regressions on dummy variables indicating the treatment and the individual-specific control variables described in Section A.1.1. Columns (1) (3) and (5) also contain Period-specific dummy variables. Standard errors are clustered by market. * $p < 0.10$, ** $p < 0.05$, *** $p < 0.01$.

Table A2: Treatment Effects for Additional Outcomes

	(1) Full Info	(2) Choice-100	(3) Choice-85	(4) Choice-60	(5) No Info
Offered Donations	0.234***	0.254***	0.249***	0.181***	0.260***
	(0.024)	(0.032)	(0.025)	(0.031)	(0.054)
Observations	1440	1440	1440	1440	1440
Cluster	12	12	12	12	12

Notes: The table shows the results from random-effects regressions of the offer on the donation level associated with the product for the five treatments. The regressions also include a set of individual-specific control variables. Standard errors are clustered by market. * $p < 0.10$, ** $p < 0.05$, *** $p < 0.01$.

Table A3: Correlations between Prices and Donations

A.1.1 Additional Control Variables

The following variables are used as additional control variables in the regressions:

- *age*: Participant's age
- *Degree*: Dummy variable indicating the degree the participant plans to obtain from the course currently enrolled in (Bachelor= 1, Master= 2, other= 0).
- *Female*: Dummy variable indicating the participant's gender.
- *German*: Dummy variable indicating whether the participant's nationality is German, or not.
- *unicef*: Do you, in principle, consider unicef an organization worthy of support? (scale from 1 to 7 where 1="yes, very" and 7="no, not at all". 1 is omitted category)
- *donation frequency*: Have you donated money to charity in the last twelve months? (where 1="yes, weekly", 2="yes, monthly", 3="yes, from time to time", 4="yes, at least once", and 5="no". 5 is omitted category)
- *donation size*: If yes, how much did you donate approximately in the last year? (in Euro)

A.2 Dominated Choices

	Full Info	Choice-100	Choice-85	Choice-60	No Info
Dominated Choices	52/717<br>(0.0725)	55/518<br>(0.1062)	11/355<br>(0.0310)	6/210<br>(0.0286)	—
Price-dominated Choices	—	7/117<br>(0.0598)	11/257<br>(0.0428)	48/347<br>(0.1383)	220/718<br>(0.3064)

Notes: The table shows dominated and price-dominated choices, separately by treatment. For the first row (dominated choices), we consider all cases where a buyer had at least two product with visible donations to choose from and chose one of these. A choice is classified as dominated if for the chosen product (p_i, d_i), there is another product (p_j, d_j) with a visible donation and $p_i \geq p_j$ and $d_i \leq d_j$ with at least one inequality strict. For the second row (price-dominated choices), we consider all cases where a buyer had at least two products with hidden donations to choose from and chose one of these. A choice is classified as price-dominated if for a chosen product $(p_i, \cdot)$, there is another product $(p_j, \cdot)$ with a hidden donation and $p_i > p_j$. In both cases, the first number denotes the number of (price-)dominated choices among all applicable cases. The number in brackets denotes the share of (price-)dominated choices.

Table A4: Dominated Choices by Treatment

A.3 Non-parametric Tests

	Full Info	Choice-100	Choice-85	Choice-60	No Info
Donations (offered)	61.72 (23.34)	44.50 (26.66)	31.88 (13.53)	42.85 (18.98)	14.97 (13.13)
Donations (sold)	62.10 (27.21)	43.60 (29.98)	27.29 (16.36)	40.08 (19.85)	11.51 (12.52)

	p-value (Donations, offered)	p-value (Donations, sold)
Full Info vs. Choice-100	0.119	0.166
Full Info vs. Choice-85	0.002	0.003
Full Info vs. Choice-60	0.053	0.0496
Full Info vs. No Info	<0.001	<0.001
Choice-100 vs. Choice-85	0.326	0.225
Choice-100 vs. Choice-60	0.885	0.773
Choice-100 vs. No Info	0.003	0.001
Choice-85 vs. Choice-60	0.149	0.126
Choice-85 vs. No Info	0.011	0.013
Choice-60 vs. No Info	0.001	<0.001

Notes: The table reports p-values from MWU-tests for treatment differences. The left column presents results for donations for all offered products. The right column uses donations associated with sold products only. The level of independent observations are matching group averages. The top part of the table presents, for completeness, the averages, as presented in Table 1 in the main text.

Table A5: MWU-tests for treatment differences: donations

	Full Info	Choice-100	Choice-85	Choice-60	No Info
Prices (offered)	43.85 (5.66)	41.65 (12.75)	39.51 (8.08)	47.11 (9.80)	43.07 (8.08)
Prices (sold)	40.64 (6.44)	36.28 (13.68)	34.41 (9.72)	44.03 (10.70)	39.48 (8.92)

	p-value (Prices, offered)	p-value (Prices, sold)
Full Info vs. Choice-100	0.488	1
Full Info vs. Choice-85	0.043	0.0496
Full Info vs. Choice-60	0.299	0.204
Full Info vs. No Info	0.564	0.488
Choice-100 vs. Choice-85	0.773	0.564
Choice-100 vs. Choice-60	0.225	0.419
Choice-100 vs. No Info	0.624	0.954
Choice-85 vs. Choice-60	0.043	0.024
Choice-85 vs. No Info	0.166	0.248
Choice-60 vs. No Info	0.166	0.094

Notes: The table reports p-values from MWU-tests for treatment differences. The left column presents results for all offers. The right column uses prices of sold products only. The level of independent observations are matching group averages. The top part of the table presents, for completeness, the averages, as presented in Table 1 in the main text.

Table A6: MWU-tests for treatment differences: prices

	Full Info	Choice-100	Choice-85	Choice-60	No Info
Payoff buyer	79.10 (6.33)	83.53 (13.60)	85.59 (9.71)	75.97 (10.70)	80.35 (8.93)
Payoff seller	30.79 (2.83)	31.25 (5.10)	32.71 (4.43)	35.72 (3.64)	37.68 (3.91)

	p-value (Payoff buyer)	p-value (Payoff seller)
Full Info vs. Choice-100	0.488	0.908
Full Info vs. Choice-85	0.021	0.225
Full Info vs. Choice-60	0.326	0.004
Full Info vs. No Info	0.488	<0.001
Choice-100 vs. Choice-85	0.686	0.564
Choice-100 vs. Choice-60	0.248	0.043
Choice-100 vs. No Info	0.624	0.009
Choice-85 vs. Choice-60	0.043	0.119
Choice-85 vs. No Info	0.149	0.013
Choice-60 vs. No Info	0.166	0.204

Notes: The table reports p-values from MWU-tests for treatment differences. The left column presents results for buyer payoffs, the right column for seller payoffs.. The level of independent observations are matching group averages. The top part of the table presents, for completeness, the averages, as presented in Table 1 in the main text.

Table A7: MWU-tests for treatment differences: payoffs

A.4 Additional Figures

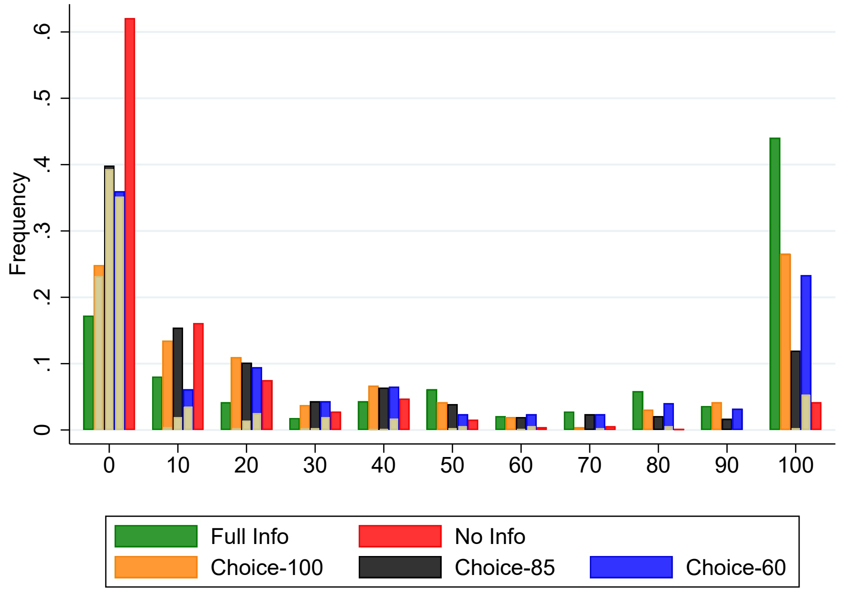

Notes: Figure plots a histogram of the donations generated by the purchase decisions of the buyers for each of the five treatments. In the Choice treatments, shaded bars denote hidden donations.

Figure A1: Histograms of Donations

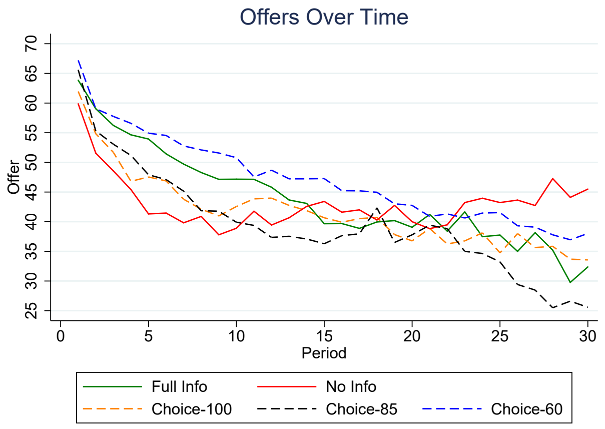

(a) Offers (incl. rejected) over Time

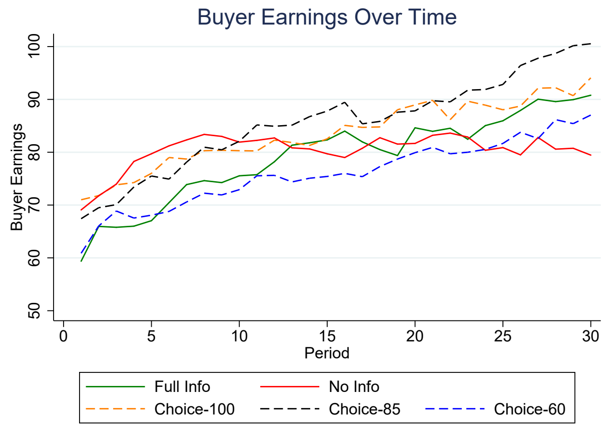

(b) Buyer earnings over Time

Notes: Figure (a) plots average offers (all offers, accepted and rejected) per period over the thirty periods of the experiment for each of the five treatments. Figure (b) plots average buyer earnings per period over the thirty periods of the experiment for each of the five treatments.

Figure A2: Offers and Buyer Earnings in the Market

A.5 Replication Study

In March 2021, we ran a replication study of Full Info and Choice-100 to address the inconclusive results regarding differences between these two conditions. As the Cologne Laboratory for Economic Research (CLER) has been closed throughout the COVID-19 pandemic, the experiment was conducted online via zTree unleashed (Duch et al., 2020), an online architecture for running zTree experiments over the internet. Like in the initial experiment, participants were recruited from the subject pool of the CLER via ORSEE (Greiner, 2015).

We sampled 28 markets per treatment condition, which provides 80% power to detect an effect of the size observed on donations in the initial experiment (0.65 sd) at the 5% significance level using Mann-Whitney-U tests. The power calculations were performed in G*Power 3.1 (Faul et al., 2009) assuming a logistic distribution as the parent distribution and performing a one sided-test. The one sided-test is justified given the directional hypothesis from the initial experiment. The achieved power is to some degree sensitive to the assumption of the parent distribution: assuming a normal distribution yields 75% power in the above specification, while a Laplace distribution yields 90% power. Two of the 56 markets had to be terminated due to technical difficulties, one in each treatment condition, so that 27 markets per treatment remain for analysis, which affects power only marginally. In the initial experiment with 12 markets per treatment condition we were powered to detect a minimal effect size of about one standard deviation (power=0.80%, $\alpha = 5\%$, MWU test, one-sided).

As performing an exact replication was not possible due to the pandemic, there are some unavoidable differences in the experimental procedures between the initial experiment and the replication, which could limit the comparability across the two experiments. Most importantly, subjects participated in the online experiment from home, so that the degree of social interactions between subjects was much lower. During the online experiment subjects joined a Zoom meeting in which the instructions for the experiment were provided and technical difficulties could be resolved. The instructions for the online experiment differed from the instructions in the laboratory only in the introductory paragraph (see Appendix B.3). Subjects did not have to turn on their camera while in the Zoom meeting. In that way the online experiment was arguably more anonymous compared to the laboratory. Payments were made via PayPal instead of cash payments used in the laboratory experiment. On average, the online experiment also took around 30 minutes more time than the laboratory experiment.

To conduct the online replication, we also had to make a number of changes to the initial zTree program. First of all, we had to add screens to collect the PayPal addresses of sub-

jects. Secondly, we had to implement a procedure to handle attrition of subjects. In case the internet connection to a subject was lost, they had 3 minutes time to reconnect to the experiment. Otherwise, the experiment was terminated for all subjects in the respective market. As mentioned above, this happened only in two markets, one per treatment condition. Last, we had to make a number of minor graphical adjustments.

A.5.1 Results - Replication Only

Summarizing our results, we find that the level of donations, prices and earnings do not differ significantly between Full Info and Choice-100. Hence, the online replication provides unambiguous evidence for Prediction 2. In what follows we describe the main findings in more detail.

Average donations in Full Info amount to 41.25, compared to 42.54 in Choice-100 ($p = 0.959$, MWU-test, see Table A9 for a complete overview of all tests). Figure A3a shows that the histograms of the realized donations and the level of donations over time exhibit no noticeable differences between the two treatments.

The prices and profits over time depicted in Figure A4a and A4b show a similar trend, just that both outcomes tend to be slightly higher in Choice-100 in the first half of the experiment. In the second half of the experiment the prices and profits in the two treatments converge. Average prices in Choice-100 are given by 35.19, while average prices in Full Info are 31.83 ($p = 0.239$).

Also following our analysis of the main experiment, we compare the quality of decisions of subjects between the online experiment and the laboratory experiment. When taking the share of dominated choices as a proxy for the quality of decisions, the decision of subjects in the online experiment are clearly not worse than in the laboratory (compare Table A10 with Table A4). Hence, we find no evidence that subjects, for example, paid less attention in the online experiment compared to the laboratory experiment.

	Full Info	Choice-100
% of buyers who bought	99.9 (0.4)	99.5 (1.0)
Donations (offered)	43.5 (23.0)	42.6 (25.2)
Donations (sold)	41.2 (26.0)	42.5 (29.6)
% revealed	100	71.5 (18.3)
% revealed (sold only)	100	70.5 (23.4)
Prices (offered)	36.9 (10.1)	39.9 (8.9)
Prices (sold)	31.8 (11.3)	35.2 (11.2)
Payoff buyer	88.1 (11.3)	84.5 (11.1)
Payoff seller	29.2 (3.6)	30.8 (4.9)

Notes: The table reports market averages and standard deviations (in brackets) for the different treatments.

Table A8: Summary Statistics of Main Variables of Interest (Replication)

	p-value (Full Info vs. Choice-100)
Donations (offered)	0.736
Donations (sold)	0.959
Prices (offered)	0.283
Prices (sold)	0.239
Payoff (buyer)	0.229
Payoff (seller)	0.328

Notes: The table reports p-values from MWU-tests for treatment differences. The level of independent observations are matching group averages.

Table A9: MWU-tests for treatment differences (Replication)

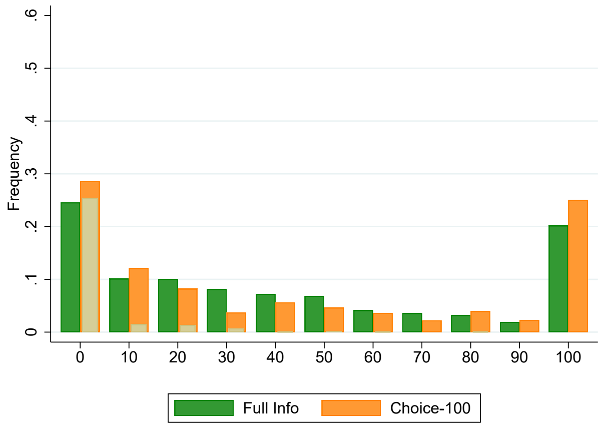

(a) Histograms of Realized Donations

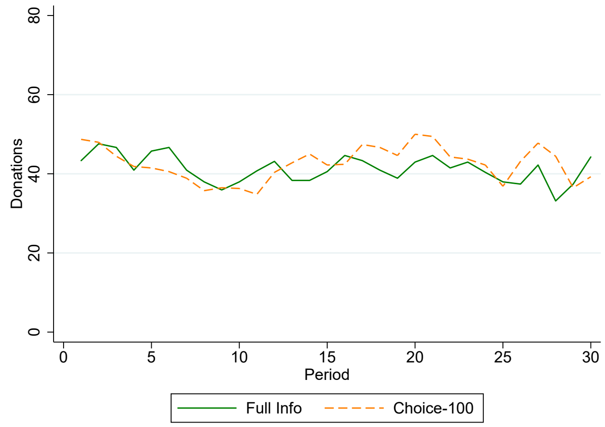

(b) Donations over Time

Notes: Figure (a) plots a histogram of the donations generated by the purchase decisions of the buyers for each of the five treatments. In the Choice treatments, shaded bars denote hidden donations. Figure (b) plots average donations per period over the thirty periods of the experiment for each of the five treatments.

Figure A3: Social Responsibility in the Market (Replication)

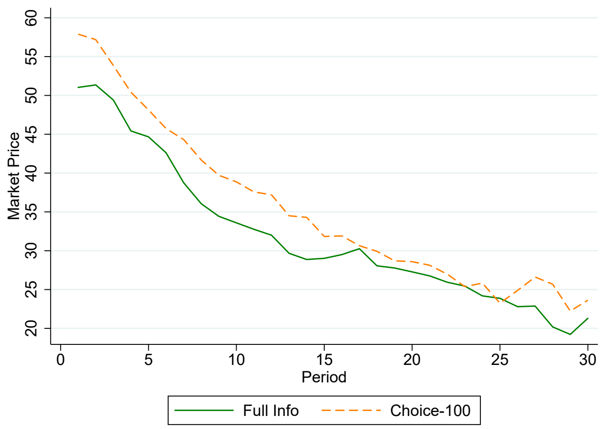

(a) Market Prices over Time

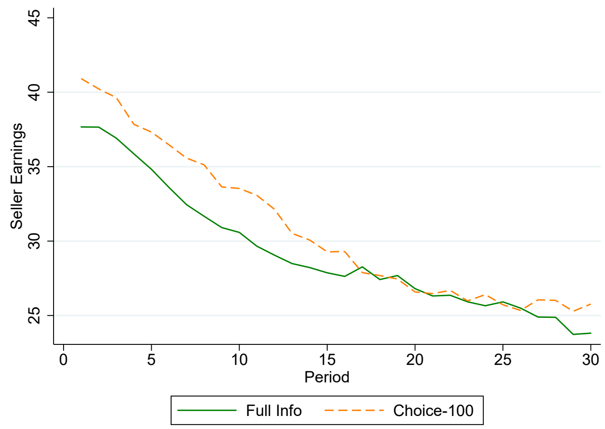

(b) Seller Profits over Time

Notes: Figure (a) plots average market prices, i.e., accepted offers, per period over the thirty periods of the experiment for each of the five treatments. Figure (b) plots average seller earnings per period over the thirty periods of the experiment for each of the five treatments.

Figure A4: Prices and Profits in the Market (Replication)

	Full Info	Choice-100
Dominated Choices	52/1,618	69/1,079
	(0.0321)	(0.0639)
Price-dominated Choices	—	18/320
		(0.0563)

Notes: The table shows dominated and price-dominated choices, separately by treatment. For the first row (dominated choices), we consider all cases where a buyer had at least two product with visible donations to choose from and chose one of these. A choice is classified as dominated if for the chosen product (p_i, d_i), there is another product (p_j, d_j) with a visible donation and $p_i \geq p_j$ and $d_i \leq d_j$ with at least one inequality strict. For the second row (price-dominated choices), we consider all cases where a buyer had at least two products with hidden donations to choose from and chose one of these. A choice is classified as price-dominated if for a chosen product $(p_i, \cdot)$, there is another product $(p_j, \cdot)$ with a hidden donation and $p_i > p_j$. In both cases, the first number denotes the number of (price-)dominated choices among all applicable cases. The number in brackets denotes the share of (price-)dominated choices.

Table A10: Dominated Choices by Treatment (Replication)

A.5.2 Results - Combining Replication and Main Experiment

	Donations (1)	Price (2)	Seller Earnings (3)	Offered Donations (4)	Buyer Earnings (5)
Choice-100	-3.557	0.320	1.278	-6.388	-0.433
	(5.659)	(2.140)	(0.910)	(5.272)	(2.142)
Choice-85	-30.049***	-5.267	2.306	-25.281***	5.353
	(7.514)	(3.272)	(1.454)	(6.595)	(3.259)
Choice-60	-19.541***	4.166	5.147***	-13.874*	-4.125
	(7.571)	(3.312)	(1.271)	(7.310)	(3.311)
No Info	-47.586***	0.295	7.538***	-41.930***	-0.457
	(6.849)	(3.180)	(1.319)	(6.132)	(3.209)
replication	-19.455***	-5.094*	-1.316	-7.283	4.845*
	(6.875)	(2.794)	(1.217)	(6.227)	(2.776)
Observations	6840	6823	13680	13680	6840
Cluster	114	114	114	114	114

Notes: The table shows the results from random-effects regressions on dummy variables indicating the treatment, a dummy indicating data collected in the replication and individual-specific control variables described in Section A.1.1. Each regression also contains Period-specific dummy variables. Standard errors are clustered by market. The regressions include all observations from the initial experiment and the replication. * $p < 0.10$, ** $p < 0.05$, *** $p < 0.01$.

Table A11: Treatment Effects on Donations - Main Experiment and Replication

In Table A11 we present the results of a regression using both the data from our main experiment as well as from the replication study. In the main text, we discussed in detail the results of Column (1) of Table A11, that is the treatment effects regarding donations. In the following we show that for the treatment effects on prices (Column (2)) and seller earnings (Column (3)) the results are very similar compared to the regression results in Table A1, i.e., using only the data from the main experiment.

In particular, to highlight some noteworthy cases:

- Market prices are significantly higher in Choice-60 than in Choice-85 (treatment

effect: 9.43, $p = 0.011$).

- Seller earnings in Choice-60 are significantly higher than in Full Info (treatment effect: 5.15, $p < 0.001$) and than in Choice-100 (treatment effect: 3.87, $p = 0.006$).
- Seller earnings in Choice-85 are neither significantly different from seller earnings in Full Info (treatment effect: 2.31, $p = 0.112$) nor than seller earnings in Choice-100 (treatment effect: 1.03, $p = 0.510$).

A.6 Additional Treatment

As discussed in the main text in footnote 9, we implemented one additional treatment, similar to CHOICE-100 as a robustness check. In this treatment, which we call CHOICE-100-COSTLY, we implement a donation schedule $\tilde{c}(d)$ which makes all *positive* donations 3 points cheaper, while no donation remains costless, i.e.,:

$$\tilde{c}(d) = \begin{cases} c(d) - 3 & \text{if} \quad d > 0 \\ 0 & \text{if} \quad d = 0 \end{cases}$$

At the same time, disclosing the donation associated with a product no longer is costless, sellers incur a cost of 3 points.

This implies that whenever a seller decides to disclose and chooses a non-zero donation, the cost of this action is the same in CHOICE-100 and CHOICE-100-COSTLY. The only difference occurs in the case where a seller decides to choose a positive donation but not to disclose. This is cheaper (by 3 points) in CHOICE-100-COSTLY.

Our motivation for running this treatment was as follows: Compared to CHOICE-100, a seller in CHOICE-100-COSTLY who cares sufficiently strongly about offering socially responsible products might be willing to offer products with high donations but wants to save on the disclosure cost. She might, for example, rather reduce the price by three points in order to make the product more attractive to a buyer. If buyers hold the belief that such sellers exist, they might be more willing to buy products with undisclosed donations. This, in turn, increases the incentives for all sellers to offer products with hidden donations, exploiting the belief of the buyers by choosing donations of zero.

We thus predict that if such a mechanism of changing beliefs about hidden donations plays a role, the share of undisclosed offers would be higher in CHOICE-100-COSTLY compared to CHOICE-100. Moreover, donations should be lower due to the exploitation effect described above.

Table A12 shows the main outcomes of interest and compares them to the CHOICE-100 treatment. The share of undisclosed offers drops slightly, by a statistically insignificant 11 percentage points ($p = 0.248$, MWU-test), while donations, if anything increase by about 11 percent, even though this difference is also far from being significant ($p = 0.419$, MWU-test). There is no difference between the treatments for any of the relevant outcome variables. Hence, this—somewhat subtle—treatment variation does not lead to meaningful changes in market behavior. To the extent that any treatment differences could, at least in part, have been attributed to biased beliefs of buyers about hidden donations, our results of this

robustness check are in line with our interpretation when comparing Choice-100 with Full Info: Buyers in our experiment seem to hold rational beliefs when inferring donations from hidden offers.

	Choice-100	Choice-100-Costly	p-value
Share buyers who bought	0.997 (0.01)	0.996 (0.014)	0.615
Donations (offered)	44.50 (26.66)	50.51 (25.98)	0.419
Donations (sold)	43.60 (29.98)	47.90 (30.92)	0.729
% revealed	0.7465 (0.13)	0.6326 (0.26)	0.248
% revealed (sold only)	0.7561 (0.17)	0.5955 (0.32)	0.157
Prices (offered)	41.65 (12.75)	38.71 (10.75)	0.525
Prices (sold)	36.28 (13.68)	33.37 (12.68)	0.773
Payoff buyer	83.53 (13.60)	86.40 (12.93)	0.773
Payoff seller	31.25 (5.10)	29.41 (4.18)	0.356

Notes: The table reports market averages and standard deviations (in brackets) for the different treatments. The final column denotes the p-value from a MWU-test, testing for differences between the two treatments. The level of observation is market averages.

Table A12: Summary Statistics of Main Variables of Interest

B Experimental Instructions

— Translated from German into English —

B.1 Treatments No Info and Full Info

Welcome To Our Experiment

During the experiment, you are not allowed to use mobile phones or communicate with other participants. Please use only the computer's programs and functions intended for this experiment. Please do not talk to other participants. Should you have a question, please raise your hand. We will then come to your desk and answer your question in private. Please do not ask your question loudly. If the question is relevant to all participants, we will repeat the question and answer it. Anybody violating these rules will be excluded from the experiment and the payment.

In addition to the 4 EUR which we will pay you simply for your participation, you can earn a substantial amount of money—how much exactly depends on your decisions. We will explain this in more detail below.

Your earnings in this experiment are calculated in points. At the end of the experiment, the amount of points which you earned, will be converted to Euros. It holds that

130 Points = 1 Euro.

General Structure

This experiment consists of 30 rounds. All of these 30 rounds are identical, which means that they follow the same rules. The points that you earn in each round will be summed up at the and for your total earnings.

In this experiment there are two different roles: buyer and seller. In addition, you will also be allocated into different groups. Each group consists of 4 sellers (Seller A, Seller B, Seller C and Seller D) and 2 buyers (Buyer X and Buyer Y). During the whole experiment, the composition of each group does not change.

At the beginning of the experiment, the computer randomly determines your role (e.g., "Buyer X" or "Seller C") and you will be informed about it on the screen. You will keep this role for the whole experiment.

Sequence of events in the individual rounds

In each round, every buyer and every seller is given an endowment of 20 points.

Decisions of the sellers

At the beginning of the round, sellers make their decisions. Every seller makes two decisions:

1. Every seller chooses a price between 0 and 120 points at which he offers his product. In case his product is bought, the seller receives this amount of points from the buyer. For a buyer who buys the product, it has a value of 100 points.

2. Additionally, every seller decides about the production cost of the product. The production cost are between 29 and 0 points. If the seller chooses the highest production cost of 29 points, 100 points will be donated to UNICEF in case the product is sold. If the seller chooses lower production cost, the donation is reduced as well. When production cost are 0, the donation is also 0 points. The table below describes the relationship between production cost and donations:

d	100	90	80	70	60	50	40	30	20	10	0
$c(d)$	29	27	25	23	21	18	15	12	8	4	0

The **earnings of a seller** are then as follows:

- If a buyer buys the offered product, the seller earns, in addition to the endowment of 20 points, the price minus the production cost.

$$\text{Earnings if product is sold} = 20 + \text{price - production cost}$$

- If the product is not bought, the seller only earns his endowment of 20 points. No production cost accrue and no donation is generated.

$$\text{Earnings if product is } \textbf{not} \text{ sold} = 20$$

It also holds that no seller can choose a combination of price and production cost which would lead to the seller making losses from selling the product.

Decisions of the buyers

After all sellers made their decisions, the buyers decide whether they want to buy one of the offered products, and if so, which one. Both sellers see on their screen the offers made

by the sellers. [No Info: I.e., they see the price that the seller wants for the product. The donation which this product generates is not visible to the buyers.] [Full Info: I.e., they see the price that the seller wants for the product and also the donation which this product generates.]

In every group one of the two buyers is chosen randomly and with equal probability. The chosen buyer then picks the one of the four products which he wants to buy, or decides to buy none of them. Then, it is the turn of the second buyer who chooses one product among the remaining ones, and this decision is then also implemented. In every round, the order is randomly determined anew.

The **earnings of a buyer** are then as follows:

- If the buyer buys one of the offered products, he earns, in addition to the endowment of 20 points, the value of the product, 100 points. From this, the price of the product is deducted.

$$\text{Earnings if product is bought} = 20 + 100 \text{ - price}$$

- If the buyer does not buy one of the offered products, he earns his endowment of 20 points. No donation is generated.

$$\text{Earnings if } \textbf{no} \text{ product is bought} = 20$$

At the end of every round, all sellers can see the offers of the other sellers and also which offers were accepted by the buyers.

The donations

The donations generated by the decisions of the participants in this experiments will be donated to UNICEF. Here, the same conversion rate of 130 Points = 1 Euro applies.

> *UNICEF, the United Nations Children's Fund, supports children in developing countries and areas of conflict. UNICEF promotes that children can go to school, receive medical care, clean drinking water as well as sufficient nutrition. Worldwide, UNICEF takes action to protect children from exploitation and abuse.* (source: unicef.de)

The full amount from this session will be donated via bank transfer by the end of today. We will email all participants a copy of the donation certificate. If you prefer not to receive this, please let us know at the end of the experiment (e.g., when you receive your payment).

B.2 Treatments Choice-100, Choice-85 and Choice-60

Welcome To Our Experiment

During the experiment, you are not allowed to use mobile phones or communicate with other participants. Please use only the computer's programs and functions intended for this experiment. Please do not talk to other participants. Should you have a question, please raise your hand. We will then come to your desk and answer your question in private. Please do not ask your question loudly. If the question is relevant to all participants, we will repeat the question and answer it. Anybody violating these rules will be excluded from the experiment and the payment.

In addition to the 4 EUR which we will pay you simply for your participation, you can earn a substantial amount of money—how much exactly depends on your decisions. We will explain this in more detail below.

Your earnings in this experiment are calculated in points. At the end of the experiment, the amount of points which you earned, will be converted to Euros. It holds that

130 Points = 1 Euro.

General Structure

This experiment consists of 30 rounds. All of these 30 rounds are identical, which means that they follow the same rules. The points that you earn in each round will be summed up at the and for your total earnings.

In this experiment there are two different roles: buyer and seller. In addition, you will also be allocated into different groups. Each group consists of 4 sellers (Seller A, Seller B, Seller C and Seller D) and 2 buyers (Buyer X and Buyer Y). During the whole experiment, the composition of each group does not change.

At the beginning of the experiment, the computer randomly determines your role (e.g., "Buyer X" or "Seller C") and you will be informed about it on the screen. You will keep this role for the whole experiment.

Sequence of events in the individual rounds

In each round, every buyer and every seller is given an endowment of 20 points.

Decisions of the sellers

At the beginning of the round, sellers make their decisions. Every seller makes three decisions:

1. Every seller chooses a price between 0 and 120 points at which he offers his product. In case his product is bought, the seller receives this amount of points from the buyer. For a buyer who buys the product, it has a value of 100 points.

2. Additionally, every seller decides about the production cost of the product. The production cost are between 29 and 0 points. If the seller chooses the highest production cost of 29 points, 100 points will be donated to UNICEF in case the product is sold. If the seller chooses lower production cost, the donation is reduced as well. When production cost are 0, the donation is also 0 points. The table below describes the relationship between production cost and donations:

d	100	90	80	70	60	50	40	30	20	10	0
$c(d)$	29	27	25	23	21	18	15	12	8	4	0

3. Every seller decides whether the buyer can see the chosen donation or not. [Choice-100: If the seller decides to make the donation visible, it appears, together with the price, on the offer screen of the buyer. If not, the buyer cannot see which donation the product generates and will also not be told later.] [Choice-85 & Choice-60: If the seller decides to not make the donation visible, it holds that:

   - The buyer cannot see which donation the product generates and will also not be told later.

   If the seller decides to make the donation visible, a random draw by the computer determines whether the donation is visible to the buyer or not:

   - With a probability of [Choice-85:85%] [Choice-60: 60%] the donation is visible to the buyer and appears together with the price on the offer screen.
   - With a probability of [Choice-85:15%] [Choice-60: 40%] the buyer cannot see which donation the product generates and will also not be told later.

   Whether the donation is visible or not, thus depends both on the decision of the seller as well as on chance.]

The **earnings of a seller** are then as follows:

- If a buyer buys the offered product, the seller earns, in addition to the endowment of 20 points, the price minus the production cost.

$$\text{Earnings if product is sold} = 20 + \text{price - production cost}$$

- If the product is not bought, the seller only earns his endowment of 20 points. No production cost accrue and no donation is generated.

$$\text{Earnings if product is } \textbf{not} \text{ sold} = 20$$

It also holds that no seller can choose a combination of price and production cost which would lead to the seller making losses from selling the product.

Decisions of the buyers

After all sellers made their decisions, the buyers decide whether they want to buy one of the offered products, and if so, which one. Both sellers see on their screen the offers made by the sellers. [Choice-100: I.e., they see the price that the seller wants for the product and also the donation which this product generates, provided the seller decided to reveal the level of the donation.] [Choice-85 & Choice-60: I.e., they see the price that the seller wants for the product and also the donation which this product generates, provided the seller decided to reveal the level of the donation and the random draw by the computer determined that the donation is visible.]

In every group one of the two buyers is chosen randomly and with equal probability. The chosen buyer then picks the one of the four products which he wants to buy, or decides to buy none of them. Then, it is the turn of the second buyer who chooses one product among the remaining ones, and this decision is then also implemented. In every round, the order is randomly determined anew.

The **earnings of a buyer** are then as follows:

- If the buyer buys one of the offered products, he earns, in addition to the endowment of 20 points, the value of the product, 100 points. From this, the price of the product is deducted.

$$\text{Earnings if product is bought} = 20 + 100 - \text{price}$$

- If the buyer does not buy one of the offered products, he earns his endowment of 20 points. No donation is generated.

$$\text{Earnings if } \textbf{no} \text{ product is bought} = 20$$

At the end of every round, all sellers can see the offers of the other sellers and also which offers were accepted by the buyers.

The donations

The donations generated by the decisions of the participants in this experiments will be donated to UNICEF. Here, the same conversion rate of 130 Points = 1 Euro applies.

> *UNICEF, the United Nations Children's Fund, supports children in developing countries and areas of conflict. UNICEF promotes that children can go to school, receive medical care, clean drinking water as well as sufficient nutrition. Worldwide, UNICEF takes action to protect children from exploitation and abuse.* (source: unicef.de)

The full amount from this session will be donated via bank transfer by the end of today. We will email all participants a copy of the donation certificate. If you prefer not to receive this, please let us know at the end of the experiment (e.g., when you receive your payment).

B.3 Online Replication of Full Info and Choice-100

Welcome To Our Experiment

As a first step, please close all programs and tabs that are not required for the participation in this experiment. In case you experience any problems with your internet connection during the experiment, you will have at least three minutes time to reopen the link and continue with the experiment. If you are offline for a longer time span, you will not be able to continue with the experiment. If you have any questions about the instructions, you will get the chance to ask them in our Zoom meeting after the time for reading the instructions is over. During the experiment you are not allowed to communicate with other participants.

The remaining instructions were exactly as in the laboratory experiment.
